# Essays on Clinical Medicine, Founded Especially on Observations of the Clinique of Professor M. Bufalini

**Published:** 1847-10

**Authors:** 


### ART. IV.

1. *Saggi di Clinica Medica, basati specialmente sopra le Osservazioni della Clinica del* Professor MAURIZIO BUFALINI. Compilati dal Dottor F. BINI, Ajuto alla Clinica Medica della Scuola di Complemento e Perfezionamento nell' I. E. R. Archispedale di S. Maria Nuova di Firenze, e dall Dottor GHINOZZI. Vol. I, Fasc 1, 2, 3, 4, e 5 ; Vol. II, Fasc 1 e 2.—*Firenze,* 1843-5.

*Essays on Clinical Medicine, founded especially on Observations of the Clinique of* Professor M. BUFALINI. Compiled by Dr. F. BINI, Clinical Assistant of the School of Completion in the large Hospital of Florence, and by Dr. C. GHINOZZI.—*Florence,* 1843-5.

2. *La Clinica Medica del* Professore G. A. GIACOMINI. Esposizione compendiata del Dottore G. B. MUGNA.—*Padova,* 1836.

*A Short Dissertation on the Clinical Medicine of* Professor G. A. GIACOMINI. By Dr. G. B. MUGNA.—*Padua,* 1836. 8vo, pp. 147.

WE have placed the above works together with the view of contrasting the practical results of two opposite systems of medicine, both tested in the clinical wards of large Italian hospitals, under two physicians who are regarded by their fellow-countrymen as the rival leaders of what may be termed the vital and the organic schools, assisted by students and junior physicians, to whom we are indebted for this record of their observations.

We reserve for another opportunity any examination of the theories or doctrines of Bufalini and Giacomini, but may now state that the former is a principal opponent of the Brownian doctrine of excitability which obtained such favour in Italy, and a supporter of the theory of the local origin of all diseases ; all constitutional changes resulting from material or organic alteration in some part to which it is necessary to direct the treatment. Giacomini also is an opponent of Brown, but on different grounds ; regarding most diseases as an increase of vital activity generally manifested under the type of inflammation. He combats the humoral pathology, and his commentator Mugna perfectly agrees with his views as to the inflammatory origin of disease, regarding delirium tremens as encephalitis, neuralgia as *neurilemitis,* tubercular phthisis as nothing more than chronic pneumonia, fevers as gastro-enteritis or arteritis; hectic fever, rheumatism, gout, chlorosis are all chronic arteritis; intermittent fever is intermittent subacute arteritis, and the constitutional irritation which follows severe operations is a diffusion of the inflammation from the divided ends of the vessels. His practice is in perfect accordance with his theory, bloodletting being his grand panacea for all human evils. The quantity of blood drawn in his wards is never less than eight ounces in the adult, more frequently fourteen or sixteen ounces. Very low diet was the general rule, barley-water or lemonade serving as ordinary beverages. On looking over the table of the cases and their treatment, which came under observation from 1830 to 1834, we were surprised at the extraordinary number of patients bled. 650 were treated, and after taking the trouble to add up the number stated to have been bled, we find this to be 504. We also added up the number in which the bleeding was repeated, and from the result it appears that 257 were bled once, 132 twice,

65 three times, 24 four times, 9 five times, 10 six times, 2 seven times, 4 eight times, 1 ten times. Besides this, leeches, calomel, tartar-emetic, and purgatives appear to have been employed with heroic vigour. Let us take a case, for example, of acute pneumonia in a labouring man, aged 33, of sanguineous temperament and strong constitution.

"The difficulty of respiration carried to the state of orthopnœa, dull deep-seated pain over the whole thorax, speech interrupted by great anxiety, mucous rattle, which never ceased, because the cough could not expel the mucus from the air-passages; dry and tremulous tongue, meteorism of the abdomen, livid countenance, eyes fixed and prominent, and the conjunctiva injected; pulse very rapid and small, skin dry and arid; all these were phenomena which announced the case to be almost desperate.

"Bleeding to 15 ounces was practised, and 2 drachms of the cherry laurel-water given. On the second day no improvement, but the orthopnœa was more severe, mental aberration, carpologia. Bleeding to a pound, and the same dose of cherry laurel-water. He died on the evening of the third day." (Mugna, p. 42.)

Injection, adhesion, and effusion of the pleuræ were discovered; hepatization of one lung and gangrene of the other. We may learn from this case, and remarks scattered through the volume, that none of the symptoms usually regarded here as expressly contradicting bleeding, were so interpreted by Giacomini. Indeed we are told that—

"Whether inflammation be acute or chronic; if suppuration have or have not taken place; if it be accompanied by marasmus, or even if it be about to extinguish life, it is still always the same, and requires the same means to subdue it; that expectoration in pulmonary diseases can never be assisted by stimulant remedies, but may always be promoted even in the last extremity by bleeding and antiphlogistics; that nutrition is not supported in the sick by so-called nutriments, but by the means which check the disease which causes the imperfect nutrition." (Mugna, p. 129.)

We have seen from the numbers bled how these views were carried out in practice, and our readers will doubtless look with interest for the statistical results. These are given in the annexed table:

| | |
|---|---|
| Number of patients treated in the clinical ward | 650 |
| Total number discharged | 617 |
| Total number cured | 585 |
| Number discharged into other wards, not perfectly cured | 22 |
| Number of deaths | 33 |
| Number of slight diseases, or those easily curable | 204 |
| Number of severe and dangerous cases difficult of cure, incurable excluded | 409 |
| *Number of absolutely incurable diseases | 15 |
| Mortality per cent. on the total number treated | $5\frac{1}{13}$ |
| Mortality per cent., those cases excluded which did not complete the cure in the clinical ward | $5\frac{1}{4}$ |
| Mortality per cent. upon the severe and dangerous diseases alone, the slight cases and those easily curable being excluded; the incurables also excluded | $4\frac{1}{5}$ |
| Mortality per cent. upon both severe and slight diseases, incurables excluded | $2\frac{14}{15}$ |

We regret that no similar table is appended to the work of Bufalini, in order that we might compare them together. Neither can we gather from the work any account of his treatment, except in the essays on lead colic, delirium tremens, chorea, and fevers, written by Drs. Bini and Ghinozzi, which fill up the text; but we know from the other writings of their master, and from having followed his hospital visits at Florence, that he practises

* 13 of these died on the day of admission or the day following; the two others were cases of hydrophobia.

a cautious and rational empiricism much opposed to the indiscriminate bloodletting of his rival. His pathological views also render him much more strict in his diagnosis and logical in his deductions from symptoms. This is especially seen in the care with which thoracic diseases are discriminated, while Mugna never refers to physical diagnosis, apparently establishing the character of these diseases by general symptoms alone. The total number of cases treated in the clinique of Bufalini in seven years was 1026. Of these 102 died, 732 were cured, and 192 are described as "incomplete result," that is to say, either imperfectly cured or discharged into other wards. The numbers of slight and serious cases are not given, but however considered, it appears that the per centage of deaths is much greater, and that of cures much less than in the practice of Giacomini. Probably this may be partly explained by the latter, as we should judge from the table of his cases, having selected acute diseases as a general rule for his clinical wards; Bufalini those of a chronic character, with a greater regard to their diagnosis, as illustrated by their pathology. We must also in fairness add, that much looseness appears in the manner in which diseases are stated to be *cured* in the tables of his rival. Thus we see a case of ascites cured in 10 days, cases of rheumatic fever in 3 and 4 days, peritonitis in 2 days, quartan intermittent in 6 days, hæmoptysis in 9 days, hepatitis in 6 days, and so on. Now this alone is enough to make one sceptical, when called upon to admit the extraordinary success of treatment; at the very best, if the diagnosis of the above cases was correct the result could only have been what Bufalini would style "incomplete."

We fear that we cannot carry the contrast between these teachers much farther, because each selecting his own cases for the clinique, would naturally prefer examples bearing out his own ideas. Thus, in the tables of Giacomini, a large proportion of cases are seen to be inflammatory, aud his depleting system appears to have worked well, whereas in those of Bufalini, organic diseases occur in a much greater number. Without any common starting-point, therefore, we cannot form a correct opinion of relative success. To do this it would be necessary to obtain information as to the severity of each case; the intensity and duration of the disease before and after the admission of the patient; the various complications of the disease, and the treatment before and after admission. Then, again, varieties of climate, manners, and customs, diet, and social position of the patients must be considered. This is a kind of information which it is impossible to afford in a statistical table, and that which constitutes the real difficulty in the study of medical statistics.

In another paper we have alluded to the successful treatment of pneumonia on the homœopathic system, or perhaps we may say, have there shown that the natural progress of this disease in the majority of cases is towards recovery.

We should have made a calculation of Giacomini's success with his bleedings, but as it does not appear that physical diagnosis is much practised in his wards, we might be reasoning on an unsound basis, but it may be interesting to show the result of Bufalini's more mild system—142 cases of pneumonia were treated by him. Of these the result gives 113 cures, 18 deaths, 11 incomplete result. In 82 of these cases the disease was complicated by bronchitis or pleuritis, or gastric disturbance. Of the cases of deaths, many were received when the disease was in a very advanced stage, having run from six to fourteen days without treatment. The treatment is not described, but appears to have been mildly antiphlogistic and expectant.

Of 29 cases of pleuritic effusion there resulted 19 cures, 7 deaths, 3 incomplete result. In 5 of these cases paracentesis was performed, and of these 5, two died. In most of the cases the effusion was very large, filling the whole pleural cavity. The right side was affected in 9 cases, the left in 20.

We do not find other matters of interest in either of the tables before us. The text of Dr. Mugna's work is taken up by short dissertations on inflammations of the brain and nervous system, and of the organs of the chest and abdomen. They are far below mediocrity. The essays of Dr. Ghinozzi on delirium tremens and tetanus, with an unfinished essay on fevers; and of Dr. Bini on lead colic and chorea, although creditable as theses, contain nothing worthy of translation or analysis, nor are they by any means to be considered as *clinical.*

It may be well, before concluding, to give a short account of the clinical system of the Italian Hospitals, which are, generally, very large establishments, containing from one to three thousand beds. There are four clinical wards, for the male and female medical and surgical cases. Each of these is under the direction of a clinical professor, who takes his turn of duty for a year or session. The students are obliged to attend punctually at the morning and evening visits. Each patient is consigned to one of the students, who, before the visit, obtains from the patient or his friends his name, country, condition, age, progress of the disease, stage of the disease, exciting causes, treatment that has been adopted, and present symptoms. This is all written upon paper ruled for the purpose, and when the professor or his assistant visits the patient, the student reads it, and explains the reasons of his diagnosis. Afterwards, every morning and evening, he registers the most important changes in symptoms and treatment. Before the patient leaves the hospital, the student reads his account of the case to the professor, and it is then deposited in the records of the clinical ward. If the patient dies, before the body is opened, the student reads the history of the case with the diagnosis, and remarks of the professor; and then, other students examining the various organs, he writes an exact account of what is observed, and remarks upon the points in confirmation of, or in opposition to, the diagnosis made during life. If any student refuses to attend the patient consigned to him, or does not keep the history of the case, and consign it to the clinical archives, he is not admitted to scholastic examination; while assiduity and exactness are favorably considered at his examinations, and in the distribution of honours and profit.

This system is, in the main points, similar to that introduced in Dublin from Germany, with such marked success by Drs. Graves and Stokes, and is admirably adapted for training up a school of diligent practical observers. Its general adoption here is the only mode of checking the grinding system, and transforming the student into the man of experience.

